# On the Treatment of Chronic Dysentery by Voluminous Enemata of Nitrate of Silver

**Published:** 1882-09-20

**Authors:** Stephen MacKenzle

**Affiliations:** Physician to, and Lecturer on Medicine, at the London Hospital


### ON THE TREATMENT OF CHRONIC DYSENTERY BY VOLUMINOUS ENEMATA OF NITRATE OF SILVER.

BY STEPHEN MACKENZIE, M.D., F.R.C.P., PHYSICIAN TO, AND LECTURER ON MEDICINE AT THE LONDON HOSPITAL.

In the treatment of ulceration of mucous membranes within reach of sight and touch, all practical physicians and surgeons are convinced of the great importance of local applications, whether the seat of the disease be the pharynx, the larynx, the eye, the cervix uteri, or elsewhere. In these situations, whether the ulceration be of constitutional or local origin, we employ local treatment, with or without internal medication, and as the effects of treatment can be observed, no one doubts its efficacy. All practical surgeons are assured of the beneficial influence of applications of nitrate of silver, and other mineral astringents and mild escharotics in treating inflammations of mucous membranes. How different is the treatment of dysentery from that of inflammation of the upper part of the alimentary tract. It may, of course, be remarked that treatment which is obviously beneficial to such parts as our hands can reach, cannot, on account of physical difficulties, be applied to the length of the colon. But this difficulty is not wholly real. With a view of rendering our practice in the treatment of dysentery more successful, and more in accordance with our procedures elsewhere, Dr. Horatio Wood, of Philadelphia, has suggested the use of a large enemata of nitrate of silver, so as to bathe with a solution of this salt the whole mucous membrane of the colon. This treatment he has appropriately designated "the rational treatment of dysentery." I am aware that enemata have long been employed in the treatment of dysentery, but the importance of *large* enemata has not been insisted on, and their use is not general. Ringer, Gairdner, Bristowe, Niemeyer and others recommend the employment of enemata, and, in some instances, of nitrate of silver, but none enjoin the use of such large enemata of nitrate of silver as recommended by Dr. Horatio Wood.

"The disuse of local applications in dysentery is largely, no doubt," Dr. Wood observes, "the result of our former inability to make use of applications to any other than the extreme lower portions of the colon. By the use of forced enemata, so-called, we are now, however, able to reach every part of the large intestine. In giving such injections, it should be first remembered that the name is a misnomer; that no force should ever be used. The patient should be brought to the edge of a hard bed, placed in a position somewhat resembling that for lithotomy, his buttocks resting upon a hard pillow in such a way as to elevate the pelvis, and cause the injected fluid naturally to flow downward and inward. A well-oiled, smooth, somewhat flexible, hard tube, with openings at the side (an œsophageal tube will answer well), and a closed end, must then gently and slowly be introduced from eight to twelve inches into the rectum. The free outer end of this may be connected with a Davidson's syringe, and the fluid thus be slowly pumped in. A better plan is to unite it with a flexible india-rubber tube, in the end of which a funnel is inserted. This being elevated five or six feet, the water is poured in, and by its own weight, with irresistible gentleness, forces its way into the gut. Instead of a funnel being used, the tube may be so arranged as to empty a bucket or other reservoir of water, placed five or six feet above the patient. A direct connection may be made, or the principle of the syphon taken advantage of. Finally, the so-called fountain syringe may be substituted. In any case the liquid should be about the temperature of the body, so as not to provoke peristalsis by the stimulus of heat or cold."

Dr. Wood writes that, whilst some considerations would lead us to expect variety in the character and strength of the applications that would be likely to be serviceable, his experience of throat affections led him to select nitrate of silver in the first instance, and he has been so satisfied with its results that he has employed no other. Drachm doses have, in his hands, never occasioned constitutional symptoms, and less than forty-grain doses have not accomplished much good. In one of my own cases, to be narrated, a single enema of thirty grains of nitrate of silver in three pints of water caused the complete cessation of chronic dysentery that had lasted two years. This I regard as exceptional. I believe that, as a rule, at least a drachm of nitrate of silver to three pints of water should be used, and I have employed as much as a drachm and a half of nitrate of silver to this quantity of water with good result and without danger. Dr. Wood properly discusses the possible effects of the application for a longer period than occurs elsewhere of so large a dose of nitrate of silver to an absorbent surface, but has never seen the least inconvenience arising from it. My experience is in entire accord with it. He suggests that, in case of the enema being retained, and fear arising as to the toxic effects of a large dose, a solution of common salt should be at hand to inject, and neutralize the nitrate of silver. In two cases in my absence, my house-physician thought it desirable to do so.

I, myself, have been led to try perchloride of iron instead of nitrate of silver, as the former would be wholly destitute of the dangers entailed by the use of the latter, and any absorption which took place would be an advantage rather than the reverse. But my results have not been nearly so good with iron as with silver. I have not experienced any practical difficulty, inconvenience, or the least danger with these large and comparatively strong injections of nitrate of silver, whilst the results have been so encouraging that I am anxious the treatment should be tried on a larger scale, and by other observers.

It will, of course, be understood that this plan of treatment is not suitable for diseases of the intestines above the ileo-cæcal valve.—*Lancet, April 22, 1882, p. 641.*

